# Congenital Central Nervous System Malformations: A Rare Case of an Encephalocele and Literature Review of Its Associations, Imaging Modalities, Radiological Findings, and Treatments

**DOI:** 10.7759/cureus.15959

**Published:** 2021-06-27

**Authors:** Bala C Veerabathini, Kaushik Manthani, Ayesha Hussain

**Affiliations:** 1 Transitional Year Resident, Peconic Bay Medical Center-Northwell Health, Riverhead, USA; 2 Family Medicine, Peconic Bay Medical Center-Northwell Health, Riverhead, USA; 3 Maternal Fetal Medicine, Stony Brook University Medical Center, Stony Brook, USA

**Keywords:** central nervous system, malformation, congenital, encephalocele, meningocele, meningoencephalocele, cystic hygroma, lemon sign, banana sign, spalding sign

## Abstract

Congenital central nervous system (CNS) malformations are relatively rare conditions present in fetuses that may result in intrauterine fetal deaths (IUFDs). We report a case of a 42-year-old female who presented at 29 weeks gestation with lack of a fetal heart beat likely due to a congenital malformation resulting in IUFD. This case report and literature review provides a better understanding of the encephalocele as a harbinger for IUFD.

## Introduction

In the United States between 2015 and 2017, the fetal mortality rate was 587.6 fetal deaths per 100,000 live births. Some 10.8% of the fetal deaths were attributed to congenital malformations, 13.9% of the fetal deaths were due to maternal complications, 26.5% of fetal deaths were due to placenta, cords and membrane complications, 9.6% were due to maternal conditions unrelated to pregnancy, and 39.2% were due to all other/unspecified causes. Congenital malformations of the CNS include anencephaly, encephalocele, meningocele, meningoencephalocele, microcephaly, congenital hydrocephalus, reduction deformities of the brain, spina bifida, and other congenital malformations of the spinal cord and nervous system [1]. 

An encephalocele is a type of neural tube defect with a prevalence of 1 in 10,000 live births and is more common in South and Southeast Asian populations [2-3]. Embryologically, the neural tube is a narrow channel in the fetus in which the brain and spinal cord form. It folds and closes in the third or fourth week of pregnancy. Lack of closure and failure in the separation of the surface ectoderm from the neuroectoderm can lead to a defect in the skull and result in herniation of brain tissue with either a pedunculated base or a sessile base [2-4]. The herniated contents may be covered by skin or a thin membrane and may represent a small sac. Encephaloceles may affect any part of the skull but most affect the occipital area (75% of cases) and have worse outcomes than those that affect other areas of the skull. Defects containing only meninges are called meningoceles, defects containing both brain and meninges are called meningoencephaloceles, and defects containing tissue from both brain and spinal cord are called encephalomyeloceles [2-3].

The exact underlying etiology of an encephalocele is unknown, and cases are typically sporadic but thought to be due to genetic and environmental causes. Genetic causes can include chromosomal and gene abnormalities and environmental causes include deficits in maternal nutrition, toxins, or infections. Encephaloceles are also associated with a family history of neural tube defects and may also be associated with over 30 neurological conditions such as Dandy-Walker or Chiari malformation to Meckel-Gruber syndrome, Fraser syndrome, Roberts syndrome, and Walker-Warburg syndrome [2]. At least 60% of encephalocele cases were found to have associated malformations and chromosomal abnormalities [4].

The prognosis and mortality with encephaloceles depend on the location. Posterior encephaloceles have a mortality of greater than 50% and anterior encephaloceles have a mortality of about 20%. Due to herniation of brain tissue, mortality rates are relatively higher compared to other fetal abnormalities. In survivors, there is a greater than 50% likelihood of neurological complications [5].

## Case presentation

A well-appearing 42-year-old gravida 5 para 4 woman with no significant past medical or surgical history presented to the hospital at 29 weeks gestation determined by ultrasound with a chief complaint of no fetal movement for one day. Of note, she did not have any routine prenatal care and did not take any medications including prenatal vitamins during her pregnancy. Her last menstrual period corresponded to an estimated gestational age of 30 weeks. The physical exam was unremarkable, including a benign cardiovascular, respiratory, abdominal, neurological, and musculoskeletal exam.

Her initial vitals were notable for an elevated blood pressure of 168/100, but otherwise vital signs were within normal limits. The fetal ultrasound dimensions are noted in Table 1 and pertinent lab results on admission are listed in Table 2. She denied any tobacco, alcohol, or recreational drug use. Urine drug screen and severe acute respiratory syndrome coronavirus 2 (SARS-CoV-2) Abbott (dry swab) were negative. The estimated gestational age by ultrasound was 29 weeks and the estimated fetal weight by ultrasound was 1335 g.

**Table 1:** Fetal dimensions on ultrasound and corresponding fetal age.

Dimension	Length (cm)	Corresponding fetal age
Biparietal diameter	7.1	28 weeks, 3 days
Head circumference	27.7	30 weeks, 2 days
Abdominal circumference	24.0	28 weeks, 1 day
Femur length	5.8	30 weeks, 0 days

**Table 2:** Lab results. WBC, white blood cell; PT, prothrombin time; PTT, partial thromboplastin time

	Lab value	Normal range
WBC count	11,490/mm^3^	4,500-11,000/mm^3^
Hemoglobin count	13 g/dL	12-16 g/dL
Hematocrit	37.8%	36%-46%
Platelet count	204,000/mm^3^	150,000-400,000/mm^3^
Neutrophils	82.4%	54%-62%
Lymphocytes	11.5%	25%-33%
PT	11 s	11-15 s
PTT	27.5 s	25-40 s
INR	1	<1.1

**Figure 1:**
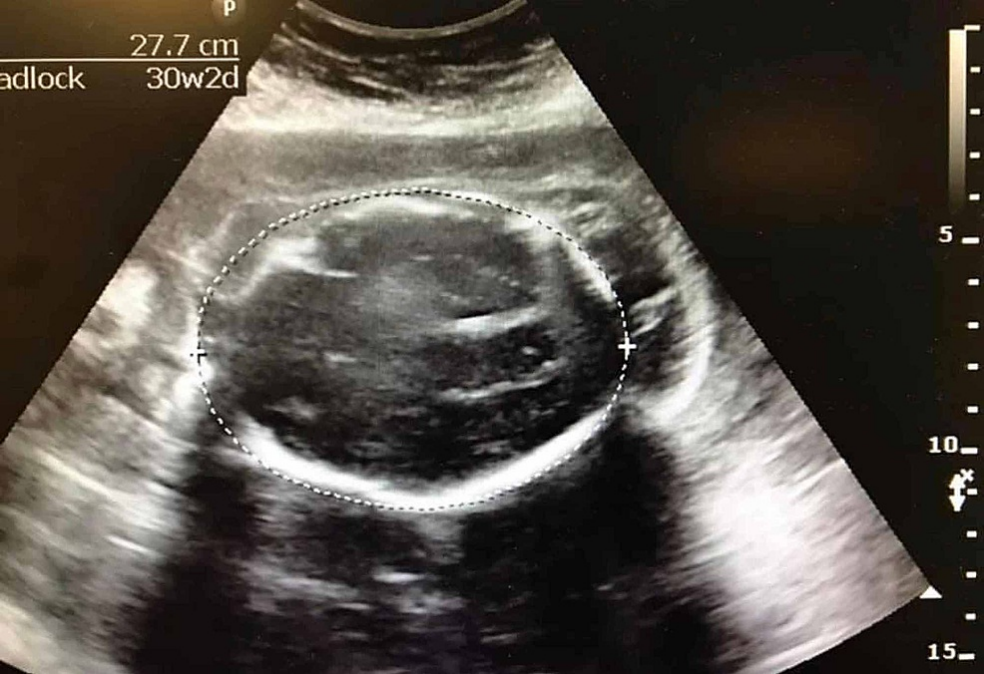
Fetal ultrasound, axial view. The dimension indicates the diameter of the head at 27.7 cm. Posterior aspect of the skull is depicted on the right side of the image.

**Figure 2:**
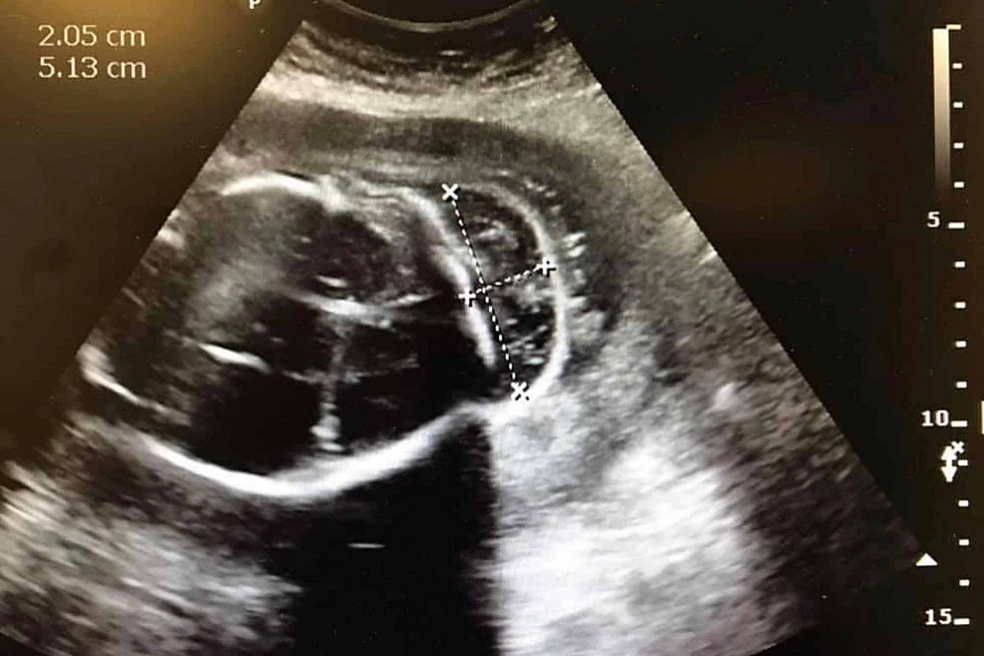
Fetal ultrasound, axial view. The dimensions indicate the size of the lesion at 2 cm by 5 cm. Posterior aspect of the skull is depicted on the right side of the image.

**Figure 3:**
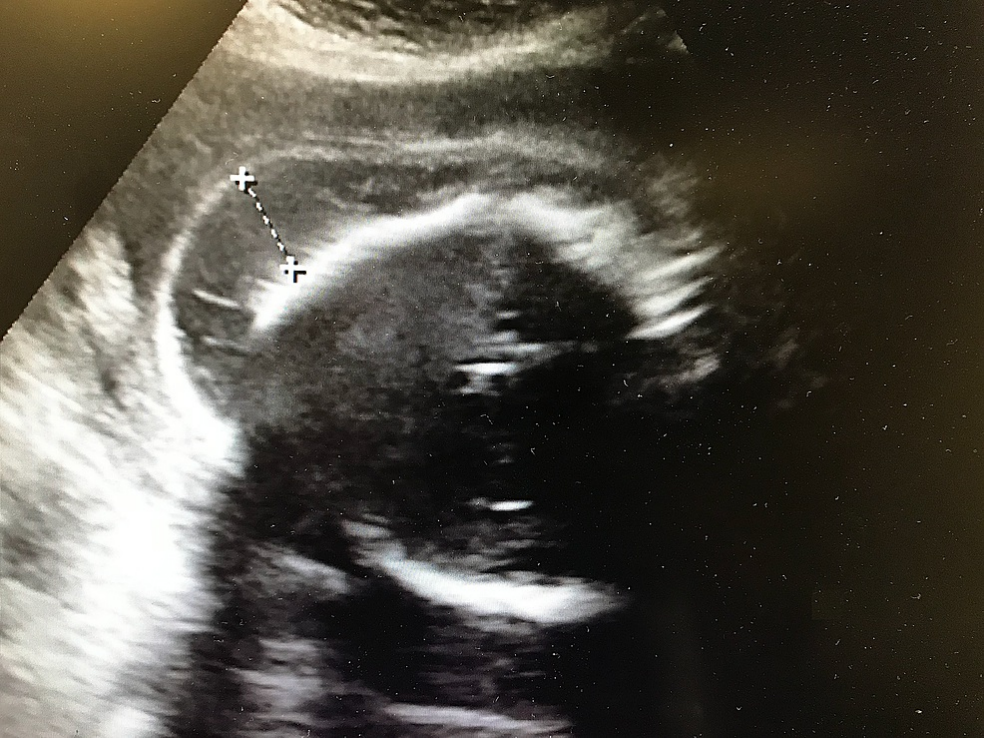
Fetal ultrasound, sagittal view. Posterior aspect of the skull is depicted on the left side of the image.

She was found to have no fetal heartbeat on fetal transabdominal ultrasound, fetal breech presentation, and posteriorly positioned placenta with a heterogeneous appearance. The amniotic fluid index (AFI) measured 6.5 cm, which was normal. There was an echogenic structure at the posterior aspect of the head and neck likely representing an encephalocele measuring 2.1 cm by 5.1 cm. Fetal ultrasound video is depicted in Video 1 and fetal ultrasound images are depicted in Figures 1-3.

**Video 1:** Fetal ultrasound, sagittal plane.

The patient stated she last felt fetal movements the day prior. The fetus was delivered on the same day as admission through low transverse C-section due to the breech presentation, and further complicated by the occipital mass. The fetus was pronounced non-viable on delivery and identified as male. The uterus was noted to have multiple fibroids.

## Discussion

There are several radiological findings that help in identifying an encephalocele and other abnormalities. These interpretations are dependent on fetal ultrasound, especially in third world countries with little to no access to advanced technology. Encephaloceles are most common in the occipital region of the brain as evident in Figures 1-3. It can be seen that there is a herniation of brain contents from an opening in the posterior skull which is consistent with an encephalocele (may also be considered a meningoencephalocele if meninges is also present). The head circumference and biparietal diameter may be significantly smaller than expected for fetal age. The contents of the encephalocele sac connect with the intracranial portion of the brain through the cranial defect. The sac is commonly covered by skin. The parts of the brain most frequently present in the encephalocele sac are the occipital lobes. In very low posterior encephaloceles, the cerebellum may be present in the sac as well; a normal gyral pattern may be evident depending on the gestational age. Approximately 80% of posterior encephaloceles are diagnosed during the first trimester of pregnancy [2].

An important distinction is between an encephalocele and cystic hygroma, which may appear similar. Both encephaloceles and cystic hygromas can occur with chromosomal abnormalities. While cystic hygromas are present in the posterior cervical triangle, they are known to be cystic in nature resulting from a defect in the lymphatic system. Cystic hygromas are well-circumscribed and of fluid density without any herniation of brain contents [6].

One radiological finding that can be found in fetuses with encephaloceles is the lemon sign, identified by the presence of an indentation of the frontal bone of the skull. The frontal bones lose their normal convex shape and appear flattened or inwardly scalloped (Figure 1). However, it is not specific to an encephalocele and can be present in other malformations including Chiari II malformations, spina bifida, Dandy-Walker malformations, cystic hygromas, and corpus callosal agenesis [7]. In encephaloceles associated with Chiari II malformations or spina bifida, a banana sign may also be seen in fetal ultrasounds. The banana sign refers to the banana-shaped cerebellum due to its tight wrapping around the brain stem and results from the spinal cord tethering and downward displacement of the posterior fossa contents resulting in the obliteration of cisterna magna [8]. If present, it can be difficult to discern from the provided ultrasound in this case. Lastly, the spalding sign is present in fetuses that have resulted in intrauterine fetal deaths (IUFDs). It refers to the overlapping of fetal skull bones caused by the collapse of the fetal brain and appears usually a week or more after fetal death in utero (Figure 1) [9]. 

Ultrasonography is the most commonly used form of fetal imaging. A CT or MRI was not performed for this case but may have provided confirmatory imaging for identifying encephaloceles. Table 3 shows various biostatistics for both CT and MRI with regard to encephaloceles [4]. As an MRI is good at picking up soft tissue details with higher contrast capability than CT, it may be valuable at providing exquisite details of cranial brain herniations (as evident by the higher sensitivity).

**Table 3:** Biostatistics for CT and MRI.

	Sensitivity	Specificity	Positive predictive value	Negative predictive value
CT	17%	100%	100%	46%
MRI	58%	100%	100%	24%

The fetus most likely died in the last 24 h before birth as the patient experienced fetal movements the day prior. According to a study that evaluated mortality rates for encephaloceles during a 20-year period, 76% of the deaths happened during the first 24 h after birth, and risk factors associated with increased mortality included low birth weight (<2500 g), presence of multiple defects and black race [10]. Neurosurgical interventions are available if the fetus survived birth. Anterior and posterior encephaloceles can be corrected using the pericranium to repair the dural defect. Repair of the bony defect can be done by using an autologous calvarial bone graft, an Osteopore polycaprolactone (PCL) bone scaffold filler or a titanium mesh [11]. For basal encephaloceles, where there is a defect of the skull along the cribriform plate or the sphenoid bone, a transnasal endoscopic approach can be used for hernial sac resection/hernioplasty and a concomitant transcranial approach can be used to fix the cranio-orbital region deformity [12]. For orbital encephaloceles, which may be due to a defect in the roof of the eye orbit, a surgical approach through the subfrontal route is necessary with resection of the herniated brain tissue, dural closure, and orbital roof reconstruction [13]. A cerebrospinal fluid leak is the most common postoperative complication [11]

The prognosis for fetuses with encephalocele depends on the location, size, content of the lesion, and presence of intracranial and extracranial malformations and microcephaly. Death most often occurs because of the severity of the other associated malformations or the inability to repair the defect. Posterior encephaloceles tend to be larger than other encephaloceles, with a greater amount of brain tissue involved in the lesion. Mortality is increased 3-fold in fetuses with encephaloceles associated with other anomalies compared with those with an isolated lesion [5].

## Conclusions

Encephaloceles are relatively rare in developed countries, but cases can still occur as evidenced by this case report. Access to prenatal care needs to be prioritized, especially in underdeveloped countries, to improve the screening prevalence of congenital malformations. With prenatal care and the use of multivitamins like folic acid and cobalamin, congenital malformations including encephaloceles can be decreased in incidence. Although an MRI may provide an accurate diagnosis, ultrasound remains to be an important imaging modality for diagnosing congenital deformities in pregnancies especially in resource-poor countries. Ultrasound imaging continues to be portable, safe, cost-efficient, and easy to learn and use for diagnostic imaging needs. Therefore, it is important for clinicians to be familiar with diagnostic hallmark signs of congenital malformations on imaging, especially ultrasound imaging. 
